# Genome-wide identification and characterization of circular RNA in resected hepatocellular carcinoma and background liver tissue

**DOI:** 10.1038/s41598-021-85237-y

**Published:** 2021-03-16

**Authors:** Yuki Sunagawa, Suguru Yamada, Fuminori Sonohara, Keisuke Kurimoto, Nobutake Tanaka, Yunosuke Suzuki, Yoshikuni Inokawa, Hideki Takami, Masamichi Hayashi, Mitsuro Kanda, Chie Tanaka, Goro Nakayama, Masahiko Koike, Yasuhiro Kodera

**Affiliations:** Department of Gastroenterological Surgery, Nagoya University Graduate School of Medicine, 65, Tsurumai-cho, Showa-ku, Nagoya, 466-8550 Japan

**Keywords:** Gastrointestinal cancer, Prognostic markers, Liver diseases, Tumour biomarkers

## Abstract

Circular RNA (circRNA) is a type of non-coding RNA known to affect cancer-related micro RNAs and various transcription factors. circRNA has promise as a cancer-related biomarker because its circular structure affords high stability. We found using high-throughput sequencing that seven candidate circRNAs (hsa_circ_0041150, hsa_circ_0025624, hsa_circ_0001020, hsa_circ_0028129, hsa_circ_0008558, hsa_circ_0036683, hsa_circ_0058087) were downregulated in HCC. The expression of these circRNAs was examined by quantitative PCR in 233 sets of HCC and matched background normal liver tissues, and correlations between candidate circRNA expression and prognosis were evaluated. The results of quantitative PCR showed that expression of hsa_circ_0041150, hsa_circ_0001020 and hsa_circ_0008558 was significantly lower in HCC than in background normal liver tissues. Kaplan–Meier analysis revealed that low expression of hsa_circ_0001020, hsa_circ_0036683, and hsa_circ_0058087 was associated with poor recurrence-free (RFS) and overall survival (OS) in HCC. Additionally, multivariate analysis revealed that low hsa_circ_0036683 expression was a significant prognostic factor, independent from other clinicopathological features, for inferior RFS and OS. There was no significant association between the expression of these circRNAs and hepatitis B/C status or cirrhosis. This study therefore identified circRNAs as potential prognostic markers for patients who undergo curative surgery for HCC and highlighted hsa_circ_0036683 as the most useful biomarker.

## Introduction

Recent advances in genome-wide analytical techniques have revealed that while almost all genomic lesions are transferred into RNA, as little as 2% of these encode proteins^1^. Thus, there is an abundance of non-coding RNA (ncRNA) in the eukaryotic cell. Numerous studies have uncovered important functional roles for ncRNA in regulating the interplay between DNA, RNA and protein expression^2^. Many different ncRNAs have been identified to date and they are predominantly categorized according to length^3^. Long non-coding RNAs (lncRNAs) are ncRNAs that exceed 200 base pairs and they represent a relatively abundant component of the mammalian transcriptome^4^. While lncRNAs form the biggest group of mammalian ncRNAs, circular RNA (circRNA) is a recently identified subtype of lncRNA that demonstrates greater stability than linear RNA owing to its covalently closed loop structure^5^. It is believed that circRNA regulates gene expression through various interactions with other RNA types (e.g., as sponging microRNAs) as well as through interactions with RNA-binding proteins. circRNAs may also regulate gene expression directly by influencing transcription and splicing. More recently, some circRNAs have been shown to encode proteins^6^. There is growing interest in the possible roles that circRNAs may play in the development of human diseases including malignant neoplasms. Recent studies have demonstrated abnormal circRNA expression in several types of malignancies^6^. However, the relationship between circRNA expression and cancer prognosis requires further study.

Liver cancer is predicted to be the sixth most commonly diagnosed cancer and the fourth leading cause of cancer-related death worldwide^7^. Hepatocellular carcinoma (HCC) accounts for 75–85% of primary liver cancers and is the second leading cause of cancer-related death in East Asia and sub-Saharan Africa, and the sixth most common cause in Western countries^7,8^. The main risk factors for HCC include lifestyle factors such as heavy alcohol intake, obesity, smoking, type 2 diabetes, and aflatoxin-contaminated foodstuffs, as well as background liver status including chronic hepatitis B (HBV) or hepatitis C (HCV) viral infection and associated cirrhosis^7,9,10^. Although there are several recommended treatment options for HCC, surgical resection remains the most effective therapy for prolonging patient survival^11^. However, because of complexities related to background liver status, the possibility of postoperative recurrence is higher in HCC, when compared with other gastrointestinal cancers. Consequently, the 5-year survival rate for HCC following surgery is approximately 30–57%^12–18^. Therefore, there is an urgent need to understand not only the molecular mechanisms of HCC itself, but also the molecular relationship between this disease and underlying background liver status.

In this study, we assessed the expression of circRNA in resected HCC and corresponding background normal liver tissues using a genome-wide approach. We also determined the level of circRNA in resected HCC tissues and paired normal liver tissues from patients who underwent curative HCC surgery. Expression data and clinical data were subsequently analysed to determine whether circRNA has utility as a prognostic marker in patients with HCC.

## Results

### Identification of a circRNA signature for HCC and background liver tissues using high throughput sequencing

**Figure 1:**
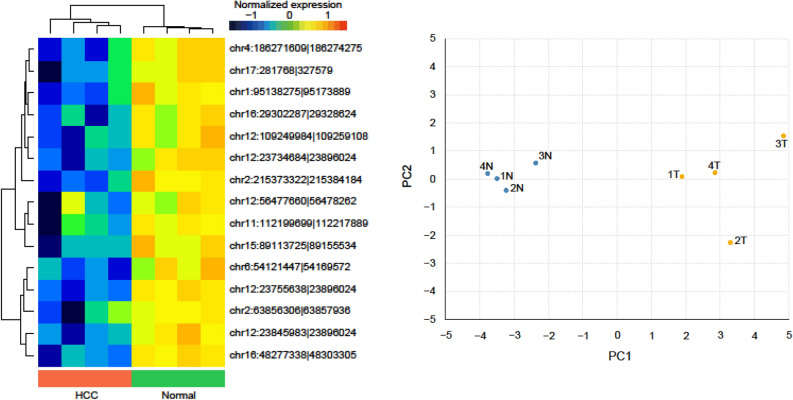
Differential expression of circRNAs between HCC and background normal liver. The figure is prepared using R software (version 3.5.3, https://www.r-project.org/)^43^ and Gplots (version 3.1.1, https://github.com/talgalili/gplots).

To develop a circRNA signature specific to HCC tumour and background normal liver tissue, we first interrogated high throughput sequencing (HTS)-based circRNA expression profiles for four cases of resected HCC. This genome-wide circRNA analysis identified 15 circRNAs that were differentially expressed between HCC and background normal liver tissue, omitting circRNAs with null expression in any of the four samples. A heatmap of the 15-circRNA signature is shown in Fig. 1, together with hierarchical clustering and principal component analysis. Eight of the 15 circRNAs corresponded to known hsa_circ IDs and we successfully generated primers for seven of these eight using CircPrimer software for the subsequent validation of the HTS data using qPCR^19^. The IDs for the seven circRNAs were hsa_circ_0041150, hsa_circ_0025624, hsa_circ_0001020, hsa_circ_0028129, hsa_circ_0008558, hsa_circ_0036683, and hsa_circ_0058087. According to the HTS data, these circRNAs were all expressed at a lower level in HCC tissues, when compared with paired background normal liver tissues (absolute log2 fold change > 2.0, p < 0.05, Student’s t-test).

### qPCR-based validation of HTS data using HCC patient samples

**Figure 2:**
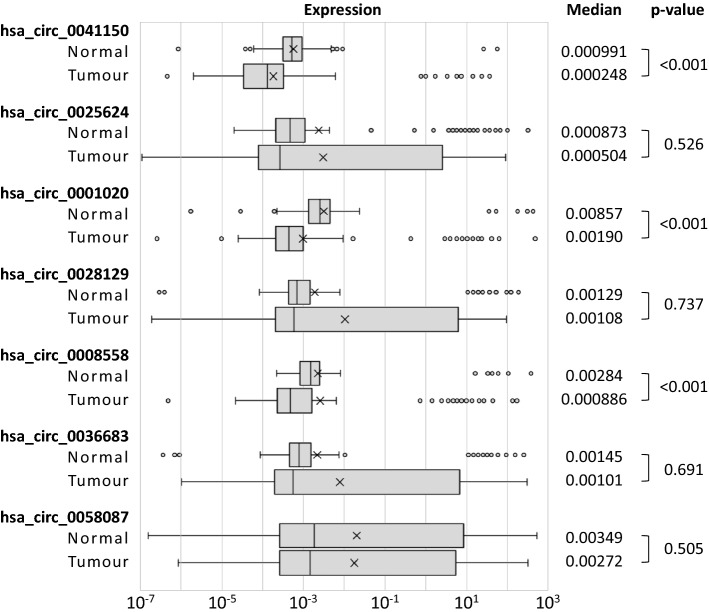
Expression analysis of selected circRNAs in HCC and background normal liver. The figure is prepared using Excel (Microsoft, 2016).

HCC and background normal liver tissues taken from 233 HCC patients who underwent liver resection, were analyzed by qPCR to determine the expression of the seven candidate circRNAs identified in the HTS analysis (hsa_circ_0041150, hsa_circ_0025624, hsa_circ_0001020, hsa_circ_0028129, hsa_circ_0008558, hsa_circ_0036683, hsa_circ_0058087). Expression profiles for these circRNAs in the collected paired samples are shown in Fig. 2. Among these seven circRNAs, the expression of hsa_circ_0041150, hsa_circ_0001020, and hsa_circ_0008558 was significantly lower in HCC tissues than in background normal liver tissues (p < 0.001 for all). These five circRNAs were therefore found to be expressed at a consistently lower level in HCC tissues, when compared with normal background tissues, in both the HTS and qPCR analyses. There was no correlation between the expression of these candidate cirRNAs in normal and tumour samples (Supplementary Fig. 1).

### circRNA expression according to background liver status

Expression of the seven candidate circRNAs according to hepatitis virus or liver cirrhosis status is shown in Supplementary Fig. 2. Regarding background normal tissues, hsa_circ_0025624 was lower in HCV-positive patients than in HBV-positive patients (p = 0.047), hsa_circ_0008558 was lower in HCV-positive patients than in HBV-positive or HBV/HCV-negative patients (p = 0.012 and < 0.001, respectively), and hsa_circ_0036683 was lower in HCV-positive patients than in HBV/HCV-negative patients (p = 0.042). Regarding HCC tissues, hsa_circ_0041150 was lower in HBV/HCV-negative patients than in HCV-positive patients (p = 0.020), and hsa_circ_0058087 was higher in HBV/HCV-negative patients than in HBV or HCV-positive patients (p = 0.032 and 0.017, respectively). In HBV-positive patients, hsa_circ_0041150, hsa_circ_0001020, hsa_circ_0028129 and hsa_circ_0008558 were all lower in HCC tissues than in background normal tissues (p < 0.001 for all). In HCV-positive patients, hsa_circ_0041150, hsa_circ_0001020, hsa_circ_0028129, and hsa_circ_0008558 were lower in HCC tissues than in background normal tissues (p < 0.001 for all). In HBV/HCV-negative patients, hsa_circ_0041150, hsa_circ_0001020, hsa_circ_0028129, and hsa_circ_0008558 were all lower in HCC tissues than in background normal tissues (p < 0.001, p < 0.001, p = 0.001 and p < 0.001, respectively).

hsa_circ_0001020, hsa_circ_0008558 and hsa_circ_0036683 expression in background normal tissues was lower in patients with liver cirrhosis than in patients without cirrhosis (p = 0.010, p < 0.001 and p = 0.002, respectively), while expression in HCC tissues was independent of cirrhosis status. hsa_circ_0041150, hsa_circ_0025624, hsa_circ_0001020, hsa_circ_0028129, hsa_circ_0008558, and hsa_circ_0058087 were all lower in HCC tissues than in background normal tissues in patients with liver cirrhosis (p < 0.001, p = 0.005, p < 0.001, p < 0.001, p < 0.001 and p = 0.036, respectively). hsa_circ_0041150 hsa_circ_0001020, hsa_circ_0028129, and hsa_circ_0008558 were lower in HCC tissues than in background normal tissues in patients without liver cirrhosis (p < 0.001 for all).

### circRNAs are predictive of HCC patient prognosis

**Figure 3:**
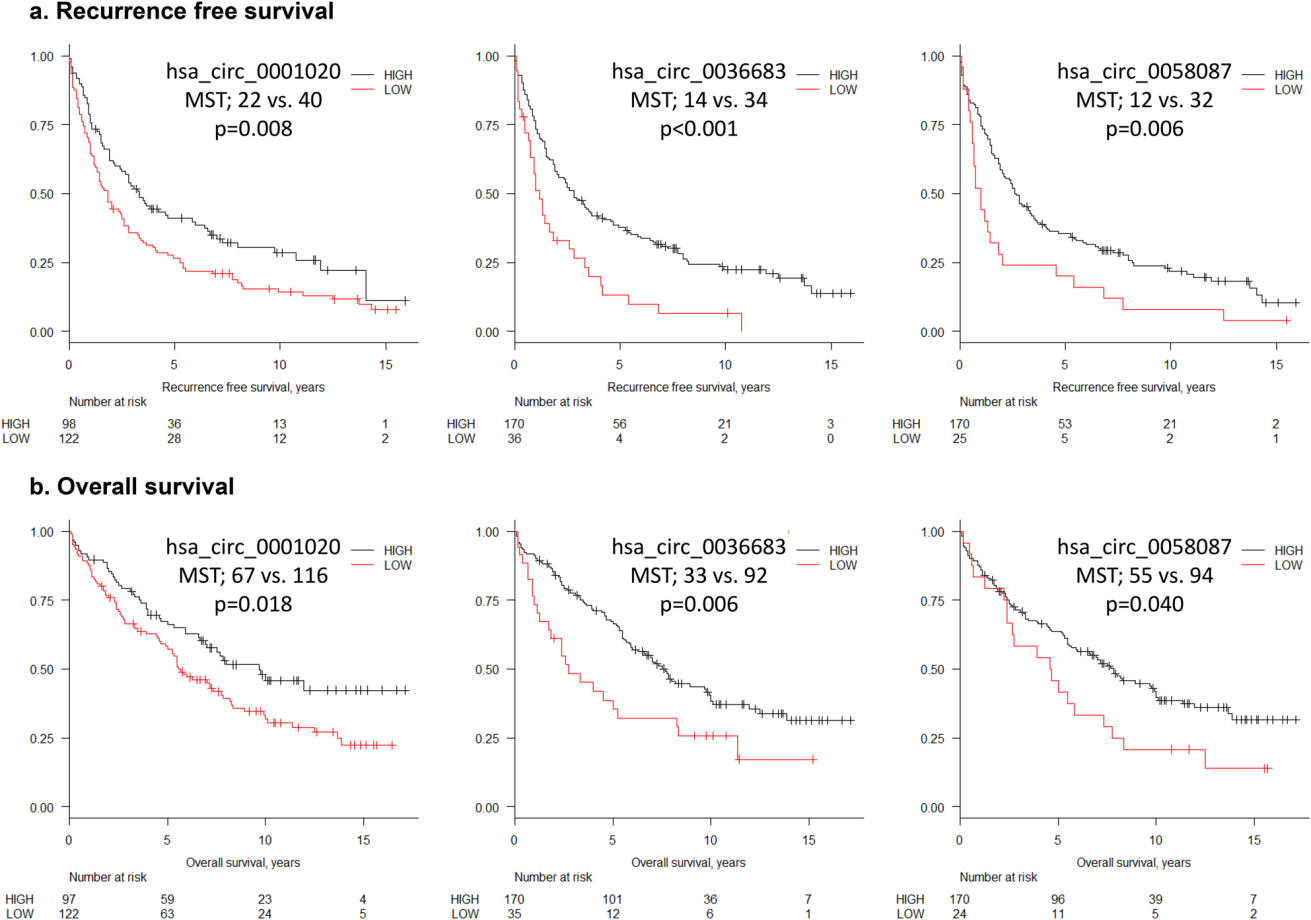
Recurrence free and overall survival based on the expression of hsa_circ_0001020, hsa_circ_0036683 and hsa_circ_0058087. (**a**) Recurrence free survival. (**b**) Overall survival. The figure is prepared using R software (version 3.5.3, https://www.r-project.org/)^43^.

Analysis of recurrence free-survival (RFS) and overall survival (OS) indicated that low expression of hsa_circ_0001020, hsa_circ_0036683, and hsa_circ_0058087 in HCC tissues was associated with a significantly inferior RFS (Fig. 3; median survival time [MST] 22 vs. 40 months, p = 0.008; MST 14 vs. 34, p < 0.001; MST 12 vs. 32, p = 0.006, respectively). Moreover, low expression of hsa_circ_0001020, hsa_circ_0036683, and hsa_circ_0058087 in HCC tissues was also associated with significantly inferior OS (Fig. 3; MST 67 vs. 116; p = 0.018, MST 33 vs. 92; p = 0.006, MST 55 vs. 94; p = 0.040, respectively). When the clinical features of the 233 HCC cases were stratified according to circRNA expression, hsa_circ_0001020 was found to be significantly associated with serum alpha fetoprotein (AFP) levels (p = 0.003), hsa_circ_0036683 (p = 0.006) and hsa_circ_0001020 (p = 0.004) with tumour size, hsa_circ_0001020 (p = 0.008) with portal vein or hepatic vein invasion, and hsa_circ_0001020 (p = 0.010) with pathological stage (Table 1).

**Table 1:** Clinical features stratified by hsa_circ_0001020, hsa_circ_0036683 and hsa_circ_0058087 expression.

Variables		All	hsa_circ_0001020 expression			hsa_circ_0036683 expression			hsa_circ_0058087 expression			MV
			Low (n = 122)	High (n = 98)	*p*	Low (n = 36)	High (n = 170)	*p*	Low (n = 25)	High (n = 170)	*p*	
Age (years)	Median (range)	65 (21–84)	64 (21–80)	65 (37–84)	0.323	64 (43–78)	65 (21–84)	0.657	67 (46–72)	65 (21–84)	0.152	0
Sex	Female	34	13	20	0.057	3	28	0.306	3	27	0.772	0
	Male	199	109	78		33	142		22	143		
Virus infection	HCV and HBV	1	1	0	0.586	0	0	1	0	1	0.718	0
	HBV	54	31	20		8	39		6	43		
	HCV	142	70	64		23	105		17	101		
	Non-HBV/HCV	36	20	14		5	26		2	25		
Albumin (g/dL)	Median (range)	3.9 (2.3–4.9)	3.9 (2.6–4.9)	3.9 (2.3–4.8)	0.927	3.9 (2.3–4.9)	3.9 (2.4–4.8)	0.693	4.0 (2.8–4.8)	3.9 (2.3–4.9)	0.985	2
Total bilirubin (mg/dL)	Median (range)	0.7 (0.2–2.3)	0.7 (0.3–2.3)	0.7 (0.2–2.3)	0.669	0.7 (0.4–2.3)	0.7 (0.2–2.0)	0.942	0.7 (0.4–1.8)	0.7 (0.2–2.3)	0.420	2
PT (%)	Median (range)	86.9 (46.9–138)	86.3 (46.5–138.0)	86.7 (59.3–132.0)	0.32	88.9 (64.0–116.1)	85.2 (46.5–132.0)	0.979	87.5 (62.8–102.7)	84.0 (46.5–138.0)	0.918	3
ICG-R15 (%)	Median (range)	11.5 (0.2–70.5)	11.5 (0.2–39.6)	11.4 (3.2–70.5)	0.677	12.8 (0.2–39.6)	11.4 (1.6–70.5)	0.175	12.8 (3.0–35.1)	11.1 (1.6–70.5)	0.426	51
AFP (ng/mL)	Median (range)	24.0 (0.8–119,923)	39 (1.2–119,923)	12.0 (0.8–36,026)	0.003	21.0 (2.1–36,978)	22.5 (0.8–43,659)	0.241	39.0 (1.9–3441)	23.0 (1.0–119,923)	0.485	3
PIVKA-II (mAU/mL)	Median (range)	44.0 (0–159,820)	49.0 (0–159,820)	39.0 (0–91,960)	0.962	126.0 (0.1–159,820)	36.5 (0–91,960)	0.027	52.0 (0.1–26,877)	34.5(0–159,820)	0.164	28
Child–Pugh classification	A	199	104	87	0.685	33	147	0.580	23	144	0.539	2
	B	32	17	11		3	22		2	25		
Liver damage	A	175	90	77	1	27	129	0.659	17	129	0.446	7
	B	51	26	21		9	35		7	37		
Tumor multiplicity	Solitary	172	91	74	1	27	130	0.832	22	120	0.091	0
	Multiple	61	31	24		9	40		3	50		
Tumor size (cm)	Median (range)	3.6 (0.15–15)	4.4 (1.0–15)	3.2 (0.15–12)	0.004	5.0 (1.0–15)	3.5 (0.15–12)	0.006	4.3 (1.5–9)	3.5 (0.15–15)	0.933	73
Differentiation	Well or moderate	212	10	5	0.426	32	12	0.726	21	159	0.209	6
	Poorly	15	110	93		3	157		3	10		
Growth form	Expansive	193	101	81	0.851	28	141	0.453	20	142	0.548	6
	Infiltrative	34	19	14		7	25		5	24		
Formation of capsule	Positive	166	90	68	0.547	26	121	1	20	123	0.479	0
	Negative	67	32	30		10	49		5	47		
Infiltration to capsule	Negative	93	49	37	0.781	16	67	0.571	8	67	0.658	1
	Positive	139	72	61		19	103		16	103		
Septal formation	Positive	158	89	63	0.135	23	118	1	17	118	1	4
	Negative	71	30	34		10	51		7	49		
Serosal invasion	Negative	162	81	72	0.085	25	117	0.282	21	112	0.449	23
	Positive	48	30	14		11	33		4	36		
Portal vein or hepatic vein invasion	Negative	171	82	82	0.008	22	131	0.059	17	128	0.465	0
	Positive	62	40	16		14	39		8	42		
Surgical margin	Negative	179	92	76	0.743	25	128	0.520	19	125	1	4
	Positive	50	28	20		10	39		6	41		
Stage	I	33	14	19	0.010	3	26	0.186	3	27	0.549	3
	II	110	51	55		15	87		14	75		
	III	59	38	15		11	39		7	44		
	IV	28	17	9		7	16		1	22		

*n* number, *HBV* hepatitis B virus, *HCV* hepatitis C virus, *PT* prothrombin time, *ICG-R15* retention rate of indocyanine green 15 min after administration, *AFP* alpha-fetoprotein, *PIVKA-II* protein induced by vitamin K absence or antagonist-II, *MV* missing value.

### Univariate and multivariate analysis of prognostic factors associated with RFS and OS in HCC

Univariate analysis revealed that pathological stage (≥ III), hsa_circ_0001020 expression (low), hsa_circ_0036683 expression (low), and hsa_circ_0058087 expression (low) were all significant predictors of inferior RFS. When multivariate analysis was performed hsa_circ_0036683 expression (low) and hsa_circ_0058087 expression (low) were identified as independent predictive factors for inferior RFS (Table 2).

**Table 2:** Univariate and multivariate cox proportional hazards regression analysis for recurrence free survival.

Variables		Univariate analysis			Multivariate analysis		
		HR	95% CI	*p*	HR	95% CI	*p*
Age (years)	≥ 65 vs < 65	1.061	0.793–1.420	0.689	1.156	0.814–1.643	0.418
Sex	Male vs female	1.439	0.935–2.215	0.098	1.475	0.861–2.528	0.157
AFP (ng/mL)	≥ 20 vs < 20	1.337	0.997–1.793	0.052	1.240	0.869–1.769	0.236
Child–Pugh classification	B vs A	1.249	0.834–1.869	0.281	1.296	0.775–2.166	0.323
Stage	III/IV vs I/II	1.481	1.099–1.996	0.010	1.128	0.783–1.626	0.517
hsa_circ_0001020 expression in HCC	Low vs high	1.502	1.106–2.041	0.009	1.276	0.872–1.868	0.209
hsa_circ_0036683 expression in HCC	Low vs high	2.020	1.363–2.993	< 0.001	1.661	1.046–2.637	0.031
hsa_circ_0058087 expression in HCC	Low vs high	1.819	1.174–2.818	0.007	1.706	1.063–2.736	0.027

*HR* hazard ratio, *CI* confidence interval, *AFP* alpha-fetoprotein.

Regarding OS, univariate analysis revealed that AFP (≥ 20 ng/dl), pathological stage (≥ III), hsa_circ_0001020 expression (low), hsa_circ_0036683 expression (low), and hsa_circ_0058087 expression (low) were all significant predictors of worse OS. When multivariate analysis was performed on these predictors, AFP (≥ 20 ng/dl), and hsa_circ_0036683 expression (low) were identified as independent predictive factors for worse OS (Table 3).

**Table 3:** Univariate and multivariate cox proportional hazards regression analysis for overall survival.

Variables		Univariate analysis			Multivariate analysis		
		HR	95% CI	*p*	HR	95% CI	*p*
Age, years	≥ 65 vs < 65	1.183	0.843–1.660	0.330	1.180	0.785–1.772	0.426
Sex	Male vs female	1.164	0.715–1.892	0.542	1.161	0.635–2.123	0.628
AFP, ng/mL	≥ 20 vs < 20	1.582	1.121–2.231	0.009	1.616	1.064–2.453	0.024
Child–Pugh classification	B vs A	1.420	0.912–2.209	0.121	1.504	0.849–2.662	0.162
Stage	III/IV vs I/II	1.516	1.074–2.139	0.018	1.274	0.841–1.930	0.253
hsa_circ_0001020 expression in HCC	Low vs high	1.540	1.073–2.211	0.019	1.314	0.834–2.070	0.238
hsa_circ_0036683 expression in HCC	Low vs high	1.837	1.179–2.862	0.007	1.708	1.006–2.900	0.048
hsa_circ_0058087 expression in HCC	Low vs high	1.645	1.015–2.666	0.043	1.442	0.851–2.444	0.174

*HR* hazard ratio, *CI* confidence interval, *AFP* alpha-fetoprotein.

### Synergistic effect of hsa_circ_0036683 on alpha fetoprotein, as a biomarker

Supplementary Fig. 3 shows the results of ROC analysis of RFS and OS 2 years after surgery. Regarding RFS, the combination of hsa_circ_0036683 and AFP had a higher AUC (0.63) than hsa_circ_0036683 (0.57) or AFP alone (0.59). In addition, for OS, the combination of hsa_circ_0036683 and AFP had a higher AUC (0.69) than hsa_circ_0036683 (0.62) or AFP alone (0.62).

## Discussion

RNA deep sequencing technology has revealed roles for ncRNAs in the progression of several diseases, including hepatitis, cirrhosis and liver cancer^20^. circRNA is a class of RNA that exhibits a unique single-stranded circular structure that is formed when the upstream 3′ splice site and downstream 5′ splice site join to form a covalently closed and stable continuous loop. circRNAs have been implicated in many important biological processes^21^. In this study, a genome-wide approach employing HTS was used to evaluate circRNA expression in HCC. Seven candidate circRNAs identified in the initial HTS analysis were subsequently examined in 233 sets of HCC samples to evaluate the relationship between expression and prognosis. The HTS data indicated that these seven circRNAs were all expressed at a lower level in HCC tissues, when compared with background normal tissues. Subsequent qPCR analysis using matched patient samples showed that hsa_circ_0041150, hsa_circ_0025624, hsa_circ_0001020, hsa_circ_0008558, and hsa_circ_0036683 were all expressed at a significantly lower level in HCC tissues, when compared with background normal tissues. Regarding prognosis, hsa_circ_0001020, hsa_circ_0036683, and hsa_circ_0058087 were all associated with worse RFS and OS. Moreover, multivariate analysis revealed that low hsa_circ_0036683 expression was an independent prognostic factor for worse RFS and OS.

Several circRNAs have been reported to be involved in HCC with some demonstrating elevated or reduced expression, suggesting that they may have utility as biomarkers in this disease^22,23^. It has been reported that circRNAs may contribute to the pathology of HCC through their role as micro RNA-sponges, protein-sponges, micro RNA transporters and as regulators of parental gene expression^22–24^. The altered expression of the seven circRNAs examined in this study has not previously been reported for HCC, although changes in hsa_circ_0041150, hsa_circ_0028129, and hsa_circ_0008558 expression have been reported in other carcinomas. An analysis of 20 sets of pancreatic ductal adenocarcinoma samples using microarray and qPCR revealed that hsa_circ_0041150 is downregulated in this disease^25^. Microarray data indicate that hsa_circ_0028129 is downregulated in colorectal cancer^26^, and that hsa_circ_0008558 is upregulated in both bladder cancer and oral mucosal melanoma^27,28^. Our study therefore demonstrates that these three circRNAs are implicated in the regulation of HCC in addition to these other carcinoma types.

Previous studies have examined the relationship between circRNA expression and background liver status^29^. Zhou et al. showed that expression profiles for hepatic circRNAs were significantly different between chronic hepatitis B (CHB) and normal hepatic tissues, with 99 dysregulated circRNAs identified in total^29^. Ji et al., reported the involvement of circ_0070963 in liver fibrosis^30^. Unexpectedly, this study did not reveal any significant association between circRNA expression and HBV, HCV, or cirrhosis status.

In addition to our study, others have also examined the relationship between circRNA expression and patient prognosis in HCC using qPCR. In a study of 70–200 HCC patients, high expression of circ_0021093, circ_0008450, circ_0128298, circ_0003998 and hsa_circ_0006916 was associated with unfavorable prognosis, as determined using Kaplan–Meier and multivariate analysis^31–35^. Low circ_0000567 and circ-ITCH expression was predictive of poor prognosis in a study of 134–288 HCC patients, as determined using Kaplan–Meier analysis^36,37^. In our study of 233 HCC patients we now demonstrate, using both Kaplan–Meier and multivariate analysis, that decreased expression of hsa_circ_0001020, hsa_circ_0036683, and hsa_circ_0058087 is predictive of poor disease prognosis.

Although we have identified several circRNAs as useful biomarkers and prognostic predictors for HCC, there are some limitations to this study. Firstly, the study cohort consisted of individuals from a single institution. Secondly, we did not investigate the underlying mechanisms of altered circRNA expression and how these changes may impact on prognosis by identifying potential gene or microRNA targets. This study simply evaluated circRNA expression in HCC and normal background liver tissues to examine its utility as a biomarker and prognostic factor in clinical practice.

In conclusion, the circRNAs hsa_circ_0041150, hsa_circ_0025624, hsa_circ_0001020, hsa_circ_0028129, hsa_circ_0008558, hsa_circ_0036683, and hsa_circ_0058087 were all associated with prognosis in HCC. In particular, hsa_circ_0036683, which was found to be an independent and significant prognostic factor for HCC, has potential utility as a candidate biomarker for this disease. Moreover, we believe that further functional analysis of these circRNAs may identify novel therapeutic targets for the treatment of this disease.

## Methods

### Patients and samples

A total of 233 frozen tumour specimens and paired para-tumour normal background liver tissues were collected from patients with HCC who underwent surgery at Nagoya University Hospital between January 1998 and January 2012. All fresh tissues were immediately frozen in liquid nitrogen and stored at − 80 °C until required. Patient characteristics are summarized in Table 1. After surgery, blood examinations, ultrasonography (US), and computed tomography (CT) were used to monitor all patients. In cases of possible recurrence, additional examination including contrast US, positron emission tomography-CT, and/or angiography were performed for confirmation. After a median follow-up duration of 66.6 months (range 0.3–206.2 months), 182 (78.1%) recurrences and 135 (57.9%) deaths occurred in the 233 patients. The median follow-up duration of all cases was 66.6 months (range 0.3–206.2 months). This study and all procedures were approved by the Institutional Review Board at Nagoya University and all patients provided written informed consent. All clinical investigations were conducted in accordance with the principles of the Declaration of Helsinki^38^.

### RNA isolation and genome-wide high-throughput sequencing

Total RNA was extracted from tissue samples using the Qiagen miRNeasy mini-kit (Qiagen, Hilden, Germany). Approximately 10 μg total RNA was then subject to ribosomal RNA depletion using the Ribo-Zero Gold Kit, as per the manufacturer’s instructions (Illumina, San Diego, CA, USA). The RNA fragments were then reverse-transcribed to create the final cDNA library using the ncRNA-Seq sample preparation kit (Illumina) according to the manufacturer’s recommended protocol. The prepared libraries were then sequenced on an Illumina Hiseq X ten platform (Illumina) and 2 × 100-bp paired-end reads (PE100) were generated according to the standard Illumina protocol. All procedures for circRNA sequencing were performed by BGI Genomics Services (Beijing, China). Sequencing reads containing low-quality, adaptor-polluted and a high content of unknown base (N) reads were removed using QCleaner v4.0.1 (Amelieff, Tokyo, Japan) before downstream analysis.

### Annotation of human circRNAs

CIRI^39^ was used to predict circRNA in this project and to integrate results based on the start and end position of circRNA. Burrows-Wheeler Aligner software (BWA 0.7.17, http://bio-bwa.sourceforge.net/)^40^ was used to align discovered reads to the hg38 reference genome. circRNAs that had the same start and end position within 10 bases were assigned to the same class. If a circRNA was recorded in the circBase (http://www.circbase.org/cgi-bin/downloads.cgi), the corresponding ID code was provided. circRNA was considered novel if it did not overlap with any registered circRNA in circBase.

### Estimation of tumour-specific circRNA and differential expression analysis

circRNAs detected in only HCC samples were considered to be tumour-specific. Tumour-specific circRNAs with a number of reads ≥ 6 were used in down-stream analyses. Differential expression analysis of circRNAs between HCC and background normal liver tissues was conducted using the limma package (3.38.3, https://bioconductor.org/packages/release/bioc/html/limma.html)^41^. The read count for circRNA was logarithmically transformed (log2 [count + 1]) and established linear models were assessed using the empirical Bayes method. The acquired p-value was adjusted by the Benjamini–Hochberg method.

### Validation of circRNA expression by qPCR

Total RNA was extracted from tissue samples using the Qiagen miRNeasy mini-kit and then converted to complementary DNA using M-MLV Reverse Transcriptase (Invitrogen, Carlsbad, CA, USA) for subsequent use in qPCR assays. PCR was performed using SYBR Premix Ex Taq II (Takara Clontech, Kyoto, Japan) under the following conditions: denaturing at 95 °C for 10 s, 40 cycles of denaturing at 95 °C for 5 s, and annealing/extension at 60 °C for 30 s. The SYBR Green signal was detected in real-time using a StepOne Plus real-time PCR System (Life Technologies, Carlsbad, CA, USA). The relative quantification method was used where the expression level of each gene in a sample was determined after normalization to the housekeeping control glyceraldehyde-3-phosphate dehydrogenase (GAPDH). Relative gene expression levels were determined using the comparative threshold cycle (2-ΔΔCT) method. To design PCR primers for circRNA targets, templates were generated using CircPrimer (Ver.1.2, https://www.bioinf.com.cn/)^19^ and divergent primers were designed with primer3 (ver.0.4.0, https://bioinfo.ut.ee/primer3-0.4.0/)^42^. All qPCR experiments were performed in duplicate, including the template-omitted negative controls.

### Statistical analysis

Continuous variables were expressed as the median and range, and gene expression comparisons were performed using the Mann–Whitney U, Wilcoxon singed rank, Kruskal–Wallis or Steel–Dwass tests. Categorical variables were compared using Fisher’s exact tests. The OS and RFS rate at each follow-up time point was estimated using the Kaplan–Meier method and comparisons were made using a log-rank test. The Cox proportional hazard model was used to perform univariate and multivariate analysis for RFS and OS. All statistical analyses were performed using R software (version 3.5.3, https://www.r-project.org/) ^43^. Statistical significance was set at p < 0.05, using two-tailed tests.

## Supplementary Information

Supplementary Table 1.

Supplementary Figure 1.

Supplementary Figure 2a.

Supplementary Figure 2b.

Supplementary Figure 2c.

Supplementary Figure 2d.

Supplementary Figure 2e.

Supplementary Figure 2f.

Supplementary Figure 2g.

Supplementary Figure 3.
