# Supplementary material for: Genome-wide identification and characterization of circular RNA in resected hepatocellular carcinoma and background liver tissue
Source: Sci Rep. 2021 Mar 16;11:6016. doi: 10.1038/s41598-021-85237-y (PMC7971023; doi:10.1038/s41598-021-85237-y)
Supplement: Supplementary file 1 — Supplementary Table 1. [file 41598_2021_85237_MOESM1_ESM.pdf]

Supplementary table 1. List of primers used for qPCR

Title:
Genome-wide identification and characterization of circular RNA in resected hepatocellular carcinoma and background liver tissue

Authors:
Yuki Sunagawa, MD†; Suguru Yamada*, MD, PhD; Fuminori Sonohara, MD, PhD†; Keisuke Kurimoto, MD, PhD; Nobutake Tanaka, MD, PhD; Yunosuke Suzuki, MD; Yoshikuni Inokawa, MD, PhD; Hideki Takami, MD, PhD; Masamichi Hayashi, MD, PhD; Mitsuro Kanda, MD, PhD; Chie Tanaka, MD, PhD; Goro Nakayama, MD, PhD; Masahiko Koike, MD, PhD; and Yasuhiro Kodera, MD, PhD
† These authors contributed equally to this work.

Affiliations:
Department of Gastroenterological Surgery, Nagoya University Graduate School of Medicine, Nagoya, Japan

*Corresponding author:
Suguru Yamada, MD, PhD
Department of Gastroenterological Surgery, Nagoya University Graduate School of Medicine, 65, Tsurumai-cho, Showa-ku, Nagoya, 466-8550, Japan
Tel: +81-52-744-2245; Fax: +81-52-744-2255; Email: suguru@med.nagoya-u.ac.jp

Supplementary table 1. List of primers used for qPCR

circRNA	Forward 5'-3'	Reverse 5'-3'
hsa_circ_0041150	CATGAGGCGGAATGTGATGG	AGTCACATCTCTCTTTGCTCAC
hsa_circ_0025624	GACCCTTACCCTGTTCAGCT	TCTGCTTGTCACCATGGCTA
hsa_circ_0001020	TTTTGCAAGAAAAGGGGCCT	GCTCTTGCCGAATGACTTCT
hsa_circ_0028129	CCGGTGGCATGAAAGATACA	AAGTAGGCCCCAATCCCAAT
hsa_circ_0008558	GACTGAGAGAGAAGGCGCA	AGGATCCAACACCTCAAGCA
hsa_circ_0036683	ACATCCGCACTTTCGTTGAC	AGAGTTGTACAGTTGGGCCG
hsa_circ_0058087	TGGCACTGATGAAGAACCCT	GTGTGCTCTCATGTTGTTCGT
GAPDH	TGGCAAATTCCATGGCA	CCTTCTCCATGGTGGT
