# Supplementary material for: Genome-wide identification and characterization of circular RNA in resected hepatocellular carcinoma and background liver tissue
Source: Sci Rep. 2021 Mar 16;11:6016. doi: 10.1038/s41598-021-85237-y (PMC7971023; doi:10.1038/s41598-021-85237-y)

Supplementary figure 2d. hsa_circ_0028129 expression based on the hepatitis virus and Liver cirrhosis status

Title:
Genome-wide identification and characterization of circular RNA in resected hepatocellular carcinoma and background liver tissue

Authors:
Yuki Sunagawa, MD†; Suguru Yamada*, MD, PhD; Fuminori Sonohara, MD, PhD†; Keisuke Kurimoto, MD, PhD; Nobutake Tanaka, MD, PhD; Yunosuke Suzuki, MD; Yoshikuni Inokawa, MD, PhD; Hideki Takami, MD, PhD; Masamichi Hayashi, MD, PhD; Mitsuro Kanda, MD, PhD; Chie Tanaka, MD, PhD; Goro Nakayama, MD, PhD; Masahiko Koike, MD, PhD; and Yasuhiro Kodera, MD, PhD
† These authors contributed equally to this work.

Affiliations:
Department of Gastroenterological Surgery, Nagoya University Graduate School of Medicine, Nagoya, Japan

*Corresponding author:
Suguru Yamada, MD, PhD
Department of Gastroenterological Surgery, Nagoya University Graduate School of Medicine, 65, Tsurumai-cho, Showa-ku, Nagoya, 466-8550, Japan
Tel: +81-52-744-2245; Fax: +81-52-744-2255; Email: suguru@med.nagoya-u.ac.jp

Legend:
The figure is prepared using Excel (Microsoft, 2016).

Supplementary figure 2d. hsa_circ_0028129 expression based on the hepatitis virus and Liver cirrhosis status

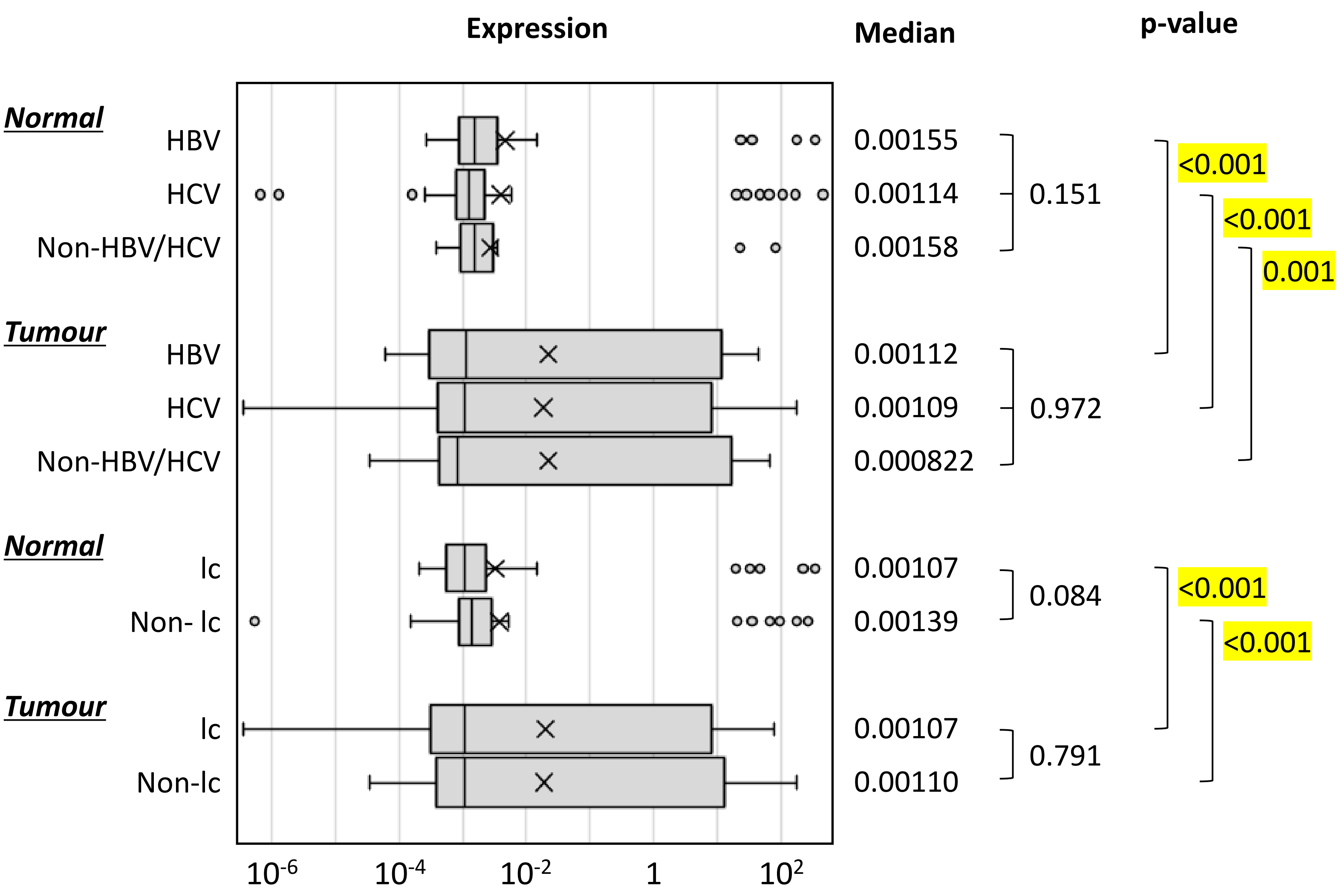

Supplement: Supplementary file 6 — Supplementary Figure 2d. [file 41598_2021_85237_MOESM6_ESM.pdf]
